# Challenges Facing the Nursing Profession in Saudi Arabia: An Integrative Review

**DOI:** 10.3390/nursrep11020038

**Published:** 2021-05-31

**Authors:** Nourah Alsadaan, Linda K. Jones, Amanda Kimpton, Cliff DaCosta

**Affiliations:** School of Health and Biomedical Sciences, Royal Melbourne Institute of Technology University, Melbourne, VIC 3083, Australia; nourah.a.s@hotmail.com (N.A.); Amanda.kimpton@rmit.edu.au (A.K.); Cliff.dacosta@rmit.edu.au (C.D.)

**Keywords:** Saudi Arabia, nursing, nursing education, nursing workforce, challenges

## Abstract

There is a paucity of recent literature identifying the issues facing the nursing profession in Saudi Arabia. The aim of this integrative review is to highlight the ongoing challenges facing the nursing profession in Saudi Arabia despite attempts to make a difference and suggests recommendations for the future. Literature published from 2000 to 2020, inclusive, relevant for nursing challenges in Saudi Arabia was accessed and reviewed from multiple sources. In Saudi Arabia, inadequate numbers of Saudi nurses have prompted an increase in recruitment of expatriate nurses. This has created its own issues including, retention, lack of competency in English and Arabic, as well as Arabic cultural aspects, insufficient experience, and a high workload. The result is job dissatisfaction and increased attrition as these nurses prefer to move to more developed countries. For national nurses, the issues are the need to recruit more and retain these nurses. There are a range of cultural factors that contribute to these issues with national nurses. There is a need to improve the image of nursing to recruit more Saudi nurses as well as addressing issues in education and work environment. For expatriate nurses there is a need for a better recruitment processes, a thorough program of education to improve knowledge and skills to equip them to work and stay in Saudi. There is also a need for organizational changes to be made to increase the job satisfaction and retention of nurses generally. Healthcare in Saudi Arabia also needs leaders to efficiently manage the various issues associated with the nursing workforce challenges.

## 1. Introduction

The population of Saudi Arabia is expected to reach 37 million by 2025 [1,2]. With the rapid growth of the Saudi population and the increasing prevalence of non-communicable diseases, such as obesity and diabetes, the recruitment, retention, training, and performance of nurses has become widely recognized as a critical issue in shaping healthcare delivery [3]. Currently, Saudi Arabia is experiencing a tremendous nursing shortage [4,5] and has such a heavy reliance on expatriate nurses, which causes unique challenges [1,5,6]. From an international context there is also a nursing workforce shortage [4,5]. So, as well as prevailing trends influencing the global nursing workforce shortage, there are some unique social and cultural considerations which is the aim of this integrative literature review.

In 1992, a Royal Decree was issued by the Saudi Arabian government to promote the Saudization policy of the nursing workforce [4,7]. This policy arose out of the realization that a continued heavy reliance on an expatriate workforce has associated risks and could precipitate a major crisis in the workforce if large numbers of expatriate nurses withdrew from the country [8]. Saudization can be defined as a policy aimed to increase the number of Saudi Arabian nationals in the workforce to gradually replace expatriate nurses with national nurses [9].

In recent years, the number of Saudi nurses has been progressively increasing with the percentage of local Saudi (relative to expatriate nurses) growing from 9% in 1997 to 27% in 2005 and 37% in 2016 [10,11]. More recently in 2018, there were 70,319 Saudi nurses which comprised around 38% of the total nurses’ population [12]. This means around 60–70% of nurses working in Saudi Arabia are expatriate or foreign (non-Saudi citizenship) and are predominantly Indian, Philippine, and Malaysian [13]. The country has made remarkable progress to increase the number of local nurses, but this progress is not readily visible and is not adequate to meet the actual need [4]. It has been forecast by 2025 the need for nurses in Saudi Arabia will have doubled [14]. These statistics mean that by 2030 approximately 100,000 nursing positions will need to be filled. As far as the nursing profession, therefore, Saudization has not achieved its goal [15]. Statistics published by the World Health Organization for 2017 show that Saudi Arabia (55/10,000) has the second highest ratio of nurses after United Arab Emirates (57/10,000), as compared to Jordan (34/10,000) which has a lower nurse population [16]. The nurse-to-patient ratio for Saudi Arabia, however, is low when compared to the international context. This nurse-to-patient ratio for Saudi Arabia in 2017 was still low when compared to United Kingdom (82/10,000), Canada (99/10,000), France (112/10,000), Australia (126/10,000), Germany (132/10,000), and United States of America (164/10,000) [16].

There are several challenges that have been associated with nursing practice advancement in Saudi Arabia. These issues will be discussed in more detail and are the focus of this paper.

## 2. Materials and Methods

To facilitate an integrative review of literature, pertinent literature was identified published between 2000 and 2021 and using the search terms “Saudi Arabia”, “nursing”, “challenges”, and “expatriate nurses”. Appropriate literature was identified from multiple sources, including searching electronic databases, and relevant reference lists. Electronic databases accessed were Scopus, Web of Science, ProQuest Social Science and Humanities, PsycCRITIQUES (Ovid), Pub Med, CINAHL, Medline, Google Scholar, Electronic Thesis, and Dissertation Systems. Documents were also accessed from official Saudi government websites. This resulted in 163 records that were reviewed with 30 duplicates removed. The second approach was to search for additional articles by manual searching to identify other pertinent literature not located in initial electronic searches. From this, the titles and abstracts were assessed with 82 excluded. A full text review of the remaining articles was undertaken by the first three authors of this paper with another 27 articles excluded. The inclusion criteria were English and peer reviewed articles about the nursing profession in Saudi Arabia. Articles were excluded if they were editorials or case reports. This resulted in 24 articles which are discussed below under the themes of Saudi and expatriate nurses’ issues.

## 3. Findings

### 3.1. Saudi Nurse Issues

One aspect to the shortage of nurses is the difficulties experienced with recruiting nationals to undertake nursing education programs. This has resulted in low levels of enrolment in nursing courses, largely due to the poor image of nursing as compared with other professions [17,18]. In a study exploring nursing education for Saudi educators and leaders, the challenges were numerous including cultural, educational, organizational reflected by weak nursing authority and lack of acknowledgement for Saudi nurses as a profession, and work challenges including poor working environments and language barriers [19]. In terms of cultural reasons this can also help explain this reticence. Saudi Arabia is a very patriarchal society with males exerting a strict code of conduct on females which they must adhere to. Prior to 1969 there were no public schools for girls and no women were employed outside of the home. Slowly, women have been permitted to become educated. Funding to support this, however, has mainly been in the areas of culturally appropriate employment, such as teachers. It is only in recent decades that funding for females to become nurses has been supported and Saudi women have actively sought employment with the limited relaxation of cultural beliefs [7]. This has resulted in 25% of the workforce and 50% of Saudi nurses being males which is in direct contrast to other nations nursing workforce [20].

The public image of the nursing profession in Saudi Arabia, however, is negative [19,21,22]. Religious and cultural issues ascribe strict gender roles in Saudi but there is limited gender segregation in nursing out of necessity. This negatively affects the nursing image because many female Saudi nurses and their families are not happy with them caring for male patients [23]. Indeed, in an exploration of the public image of the nursing profession in Saudi, more than one-third of the participants reported the gender-mixed working environment as a barrier for engagement in the nursing profession [21]. As a result of these cross-gender interactions required for nursing, there is social pressure in Saudi linked to working in these environments where practices are socially unacceptable [19]. Furthermore, strict social traditions in Saudi make it challenging for many Saudi female nurses due to the high workload as well as, until recently, the inability to drive. Furthermore, families are considered an important component of society and the framework of the identity of individuals [24]. As such it is important to maintain good relations with family members by supporting them, visiting them, celebrating with them, and showing respect for every member of the family [18]. Working in nursing is not conducive to maintaining these relationships and is considered socially unacceptable [19]. The reasons for this include such responsibilities as working weekends, night duty, public holidays, and long working days that keeps them away from home for long periods. All these factors contribute to Saudi nurses having little time to care for their families [10]. Women also tend not to choose nursing as this negatively affects their marriageable prospect because of the working conditions and needing to care for male patients, considered as inappropriate culturally due to the strict gender segregation [1,7,21]. Saudis consider marriage as a high priority and anything that hinders this is taken seriously [7]. These findings are supported in a recent study of the public image of nursing in Saudi where nearly three-quarters of the participants would be ashamed if they had a nurse in the family and less than 50% of males preferred to marry a nurse [20,21]. This further contributes to low recruitment of nationals to nursing and retention [20].

As a way of compromising work and family commitments, Saudi female nurses prefer to work in the hospital outpatient clinics only working day shifts during the week and no night duty. This is more compatible with family commitments. There is a tendency otherwise for Saudi families to request that women work only morning or afternoon shifts and no night shifts. This means, however, that the expatriate nurses must work night and weekend shifts which contributes to their dissatisfaction and retention [9].

In addition, nurses tend to receive lower wages compared to other jobs, there is a lack of professional growth, and lack of support for working mothers [10,25]. The commencing salaries for nurses are considered low for the sector at around Saudi Riyal 10,000 a month (US $2700) and there are perceptions of a lack of salary transparency [5]. Many Saudis consider nurses to be a handmaid, following a physician’s orders and uneducated, but this stereotype is slowly changing [7,26]. This is compounded by doctors themselves holding negative views of nurses [7].

Saudi men are also reluctant to choose nursing as a career because of the poor image of the profession in Saudi society and adverse comments from family and friends [7]. Nursing is viewed as women’s work, compounded by the strict gender segregation in Saudi [10]. Maybe it is because of these factors that many Saudi nurses aspire to leave the bedside clinically focused arena to become managers and educators and thereby progress their career [23]. Saudi nurses do not necessarily work for very long as a clinical or bedside nurse, they tend to apply for a scholarship to undertake their masters and/or doctoral education at an overseas university and then are appointed in a manager or education position [23]. This results in a significant proportion of the total Saudi nurses working in administration positions after minimal bedside clinical experience [27].

### 3.2. Expatriate Nurse Issues

Reliance on such a large proportion of expatriate nurses contributes to several issues, specifically, how they are recruited. These nurses are usually recruited through agencies based in countries, such as India and Philippines, with few systems or controls in place to ensure standards are followed [4]. Most expatriate nurses come from either India (26%) or Philippines (37%) as there is a ready source of nurses who are prepared to work in Saudi Arabia [3]. Contracts for recruitment are usually awarded for three years, followed by a bidding process for the next contract. This can result in a lack of continuity of contract providers and potentially contracts that are so poor that maintaining quality of personnel is difficult [3,6]. Teams of staff from Saudi Arabia consisting predominantly of doctors and administrators go to these countries to recruit nurses. Usually, nurses are not part of this recruiting team, which may result in ineffective screening issues. This can result in recruits lacking the necessary experience and being poorly matched to the positions they are recruited for [6]. Recruits are not necessarily nurses either, as credentials may be falsified to escape poverty in their own country [3].

Furthermore, new expatriate nurses may have insufficient clinical experience, with many nurses only recently qualified [10]. These nurses bring an additional workload for other experienced nurses, as the latter are required to teach and monitor the novice nurses, while still undertaking their usual duties [28]. This then contributes to a higher workload for these nurses.

Sometimes nurses are very disappointed because of workload allocation [3,6,29]. For example, medical nurses are placed in the intensive care unit or maternity unit. This is because the nurses have not been selected appropriately by the recruiting team not recruiting for the vacancies. Furthermore, the requirement for nurses is great to the point that average quality is endured, adding to the disappointment of the nurse leaders and the more talented nurses [1].

Another aspect is that often once nurses arrive in Saudi, they express dismay regarding expected workload. This is due to the level of expected responsibility their job demands which is different to what they had experienced in their own country, plus they are often newly graduated and inexperienced. In addition, nurses are assigned to take the responsibilities of the head nurse without appropriate education simply because there are not enough nurses generally [10].

There are also other issues with expatriate nurses after they have been recruited and working in Saudi. As pointed out by Mebrouk [30], most nurses in Saudi are expatriates from other countries whose values, norms, and beliefs may be different from those of the Saudi culture. Having left their home environment, these nurses try to adapt to the new environment and culture which likely affects them adversely [3,31]. The Islamic way of life is practiced by most of the population in Saudi. Consequently, Saudi nurses are preferred to care for Saudi patients because of their shared culture and language [4]. Furthermore, inadequate knowledge and cultural incompetence may cause expatriate nurses to overlook the relevance of Islamic principles in the healthcare system and patient care quality. In a study of the views of Saudi nurses, Mebrouk [30] asserts that expatriate nurses find it difficult to understand the cultural requirements of their Saudi patients. It is also reported that expatriate nurses tend to impose their own cultural norms because this is what they know [32]. This may have a detrimental effect on patients and impede their nursing care. Furthermore, it is important for foreign nurses to recognize the importance of strong extended family ties, the protection of women, an omnipotent deity, and honor [33]. Mebrouk [30] asserts that the values and beliefs of the patients and their families should be strongly taken into consideration when providing patient care. Part of the issue here also is that expatriate nurses have limited knowledge regarding Saudi Arabia cultural beliefs, specifically related to healthcare, and needs rectifying [32]. Despite these cultural and language difficulties, Saudi patients report a preference to be cared for by expatriate nurses because of their distrust of Saudi nurses and better care provided by expatriate nurses [15,34].In contrast, expatriate nurses have reported a lack of respect from Saudi patients as well as from other expatriate nurses [32].

Furthermore, this can be a stressful experience with expatriate nurses leaving their families, the culture shock of being in a Muslim country which entails strict adherence to orthodox tenets and traditions, the physical constraints of a hot climate (for example, dehydration, headaches, and exhaustion), and communication barriers [6,24,34]. Many expatriate nurses are dissatisfied with the living conditions generally. Specifically, the strict gender segregation and restricted freedom of movement of women being not necessarily welcomed [6]. In addition, there is a strict dress code for women who must cover their arms, legs, and head. Expatriate nurses may find the adjustment to these circumstances too great resulting in a high attrition rate [3,6,34].

Another complexity relating to the advancement of nursing practice in Saudi Arabiais the information exchange that is undertaken in healthcare facilities [10]. The level and type of communication are key aspects of nursing practice and patient care outcomes. Language differences between Saudis and expatriate nurses are a significant issue in terms of interaction with the nurses and the local population [35]. Most patients and their families are Saudis who speak Arabic and may or may not have English as a second language. Most expatriate are not competent in speaking Arabic [3,35]. They may also not be very competent in speaking and comprehending English which adds a further layer of complexity [34]. Expatriate nurses may, therefore, find it difficult to communicate effectively with their patients, which adds another level of complexity and dissatisfaction for these nurses [34]. Given this, Saudi nurses tend to be constantly asked to interpret for the expatriate nurses and thereby increasing their workload [6,34]. Some hospitals have a solution for this by assigning a specific person to be an interpreter for expatriate nurses, but they are not always present, causing undue distress [28]. This may also contribute to difficult professional relationships which in turn may lead to conflict, poor staff satisfaction, and high turnover [1]. These cultural and language difficulties have the potential to adversely affect nurses’ abilities to practice competently and safely [34]. In fact, expatriate nurses identified the clinical safety climate in one study as being low [32]. Other studies have identified the perceived language barrier adversely influencing patient satisfaction and compliance with medication [36].

Other factors that contribute to the high turnover of expatriate nurses relates to their accommodation and living expenses provided on site by the Ministry of Health. In the past, this provision for accommodation was extended to include family members, such as children and partners. Of recent years, this provision has been ceased and so expatriate nurses either leave their family in their home country or pay for their accommodation themselves. Generally, the nurses are on a 2-year contract which may be extended or not. It has been reported that many of these expatriates leave Saudi when they have acquired enough training and experience to work and provide their services in more developed countries, such as Canada, USA, UK, and Australia [10,32]. The average length of stay is reported to be 43 months [37]. Saudi is therefore, seen as a “stepping-stone” for some nurses to get out of their own country and impoverished situation to gain a position in a developed country. In addition, expatriate nurses are reported to leave one hospital to work in another, often a private hospital where the pay and conditions may be better than the hospital they were initially in [10]. This creates a continual process of orientating new staff who take time to gain experience and ability to function at a competent level. There is also not a strong emotional attachment to an organization consequently which has also been determined to be a strong negative predictor of turnover intent among nurses [1,6]. In a recent study investigating nursing turnover in Saudi Arabia, Filipino nurses were more likely to intend to leave their position than other expatriates and Saudi nurses. Many expatriate nurses also identified discrimination as an important contributing factor for their intention to leave. It was evident from the findings of the study that there was tension not only with supervisors from different backgrounds, but also with co-workers from different backgrounds [38].

Another factor that contributes to the high workload and dissatisfaction of nurses generally is that nurses are involved in non-nursing tasks and activities [4]. This is because there are inadequate numbers of ancillary and management personnel [10]. Hence, nurses are forced to be involved in non-clinical roles in addition to their nursing tasks, which increases their workload.

Generally, the workload for nurses working in Saudi is high due to several factors that were identified above. There are several implications of this high workload for both nurses and patients. A high workload is likely to reduce nursing care quality and increase risks to patient care outcomes [15], which can negatively impact patients’ satisfaction [39]. This may lead to a chance of increased nursing care errors and reduce the quality of nursing care and increase the length of stay [7]. Workload demands may also lead to decreased satisfaction and increased turnover. Job dissatisfaction and workload because of the effect on quality of work life, are all mentioned as determinants of turnover [3].

Nurse turnover contributes to staff shortages which in turn results in an increased workload for the remaining nurses, who are then likely to be dissatisfied and leave themselves. This higher workload also leads to absenteeism. Considerable resources are then needed to recruit and train more nurses to fill the gap and costs [27]. This nurse turnover hinders the needed expansion of the Saudi healthcare system [6,30].

## 4. Discussion

To minimize the effect of a nursing shortage in Saudi Arabia, there is an urgent need for a local nursing workforce planning strategy to recruit more national nurses and retain the current expatriate workforce. From a Saudi Arabian perspective, there is a need to improve the image of nurses and nursing to facilitate the recruitment of people into the profession. The negative social image of nursing is a barrier to joining the nursing profession for young individuals [4,19,21]. There is a need to enhance the public image of nursing to motivate the younger Saudi Arabian population to become involved in the nursing profession by providing suitable educational and employment incentives. As pointed out by Takase, Maude, and Manias [40], the negative image of nursing remains a strong factor in contributing to the international shortage of nurses generally. Increasing awareness of the value and importance of nursing is important, particularly in the popular media [10]. Nurses need to be viewed by the public as highly educated and skilled professionals dedicated to developing the profession through education and research [4].

One mechanism that can be used to enhance the public image of nursing, according to Almalki [10], is to gain cooperation from the media to increase community awareness of the importance of nursing and the vital role nurses play in the advancement of community health. In contrast to the factors that discourage people from taking up nursing in Saudi Arabia, salary, benefits, flexibility, and job security are motivations for choosing a nursing job [41]. These are all important measures that need to be considered for nurses working in Saudi Arabia.

There is also a need to upgrade existing university undergraduate nursing programs to deliver sufficient and better qualified nurses [1]. Having programs that are 5 years could be a disincentive for some to choosing nursing as a career. Currently the length of these programs at 5 years exceeds the international benchmark as most programs are either 3 or 4 years in length. Having shorter programs are more achievable and would make the qualification more attractive for potential students. In addition, nursing programs could be made more attractive by increasing the financial support for nursing students undertaking these studies [10]. Another strategy could be to pay nursing students a full salary during their intern year as opposed to the one third of a registered nurse salary they currently receive [10].

The other side to the issue regarding the shortage of nurses is the reliance on expatriate nurses, as identified earlier. Any change in policy to decrease the employment of expatriate nurses, however, could have potential international ramifications. The highest group of expatriate nurses in Saudi are recruited from the Philippines [3], who specifically educate more nurses than they need for export [42]. This is because these nurses are expected to work overseas and contribute to the country through the return of their salaries. A change in policy would therefore adversely affect the Philippines [42]. There is, therefore, a need to ensure quality of nurses in the recruitment process as well as mechanisms to address the high attrition rates.

Due to the issues identified in recruiting expatriate nurses, one solution could be to include nurses in the recruitment process. This would help ensure the quality of the nurses and ensure the right person is appointed for the right positions. Retention is much more achievable when the right person is working in the right position because it increases the satisfaction [42]. There also needs to be an emphasis on quality over quantity as the principle aim in recruiting [10]. Maybe interviewing and testing these nurses could go some way in helping to ensure this quality.

There is also a need to better integrate expatriate nurses into the work environment as well as the whole community [10]. To assist with the transition of these expatriate nurses into Saudi Arabia, there should be mandatory education courses for these nurses in Arabic, communication skills and in local culture prior to commencing their work in Saudi. Nurses are more satisfied with their position, if they have been accepted culturally and socially, especially if accepted by their families and relatives [18]. Providing this mandatory education in language and cultural aspects would assist in this process.

It may also help to ensure that the expatriate nurses have an adequate level of communication skills as part of the recruitment and selection process. Effective communication is an important organizational factor that can help solve the issue of nurse retention [1]. As discussed, despite English being compulsory in schools most Saudis do not speak English and most expatriates cannot speak Arabic. For several expatriate nurses English is also their second language, which may not be adequate [3,32]. Providing ongoing education programs would also give expatriate nurses a sense of importance and belonging. Educational opportunities for nurses have been shown to enhance nurses’ professional growth and have a significant impact on job satisfaction [24].

There are also some general strategies that could be employed to improve nurses’ satisfaction with their work environment and help improve retention. Improving workplace conditions has been identified as an important mechanism to increase retention and improve satisfaction and patient outcomes [10,27]. This could include making childcare facilities available for women onsite; flexibility in rostering a nurse caring for sick children or dependent adults [30]; allowing nurses to work part time and ensuring adequate and fair remuneration [10,27]. Other strategies could include shorter shifts and more days off to make it attractive for specifically Saudi nurses to help accommodate their family commitments. There also needs to be consideration of strategies to help reduce nurses’ workloads [10,27]. A strategy could include employing ancillary staff to decrease nurses having to undertake non nursing duties. Obviously maintaining staff levels would help considerably with nurses’ workload but better recruiting and education processes of expatriate nurses have potential to assist with nurses’ satisfaction. Involvement of nurses in decision making and having control of their environment has also been identified as a key to nurses’ satisfaction [1,6,31].

Saudi vision 2030 roadmap to reform has been specifically designed to encourage healthcare systems to improve the quality of healthcare. Improving healthcare can be obtained through the provision of an appropriately qualified nursing workforce and management, as well as appropriate staffing levels and working conditions that promotes quality of work life [6]. By implementing these various strategies identified would go towards achieving this and certainly would increase nurse satisfaction and retention and ultimately increase recruitment of Saudi nurses.

The implication of this paper and strength of this integrative literature review is that it has summarized the literature that has been written to date on the challenges that the nursing profession in Saudi Arabia is facing. A limitation is that there are not more recent articles written in this area that describes the current situation. In addition, interviewing key stakeholders to identify what they perceive are the challenges and solutions would have added to this body of knowledge. This identifies the need for further exploration of this issue.

## 5. Conclusions

Despite the remarkable development of the nursing workforce in Saudi Arabia, several challenges limit nursing advancement in this country. One of these challenges is that most nurses are expatriates despite policies aimed at increasing the number of Saudi nurses. As a result, nurse leaders face several problems, such as cultural differences, retention, and recruitment of nursing staff. These are a few of the many challenges facing the nursing profession in Saudi Arabia however, the workforce situation in Saudi is precarious due to the high dependence on foreign workers. To effectively manage these problems several strategies have been proposed including improving the public perception of the nursing profession in Saudi Arabia. In addition, there is a need to address many of the nursing issues identified in this paper to increase job satisfaction and retention overall.

## Data Availability

No data available.
